# Decoding Acute Scrotum: Diagnostic Accuracy of Ultrasound in Urgent Clinical Settings

**DOI:** 10.7759/cureus.71011

**Published:** 2024-10-07

**Authors:** Vishal Vijayakumar, Krishna Kumar Rama Krishnan, Priyadharshini Bala, Vigneshwaran S, Prabakaran T, Pooja Das

**Affiliations:** 1 Radiodiagnosis, Mahatma Gandhi Medical College and Research Institute, Sri Balaji Vidyapeeth (Deemed to be University), Puducherry, IND

**Keywords:** acute scrotum, color doppler, diagnostic accuracy, epididymo-orchitis, testicular torsion, ultrasound, varicocele

## Abstract

Background

Acute scrotum is a medical emergency commonly encountered in clinical practice, particularly in pediatric and adolescent populations. It is characterized by sudden onset of scrotal pain and may involve swelling, redness, or tenderness. The most common etiologies include testicular torsion, epididymo-orchitis, trauma, and, less frequently, tumors. Early and accurate diagnosis is critical, especially in cases of testicular torsion, where timely surgical intervention is required to preserve testicular viability. Ultrasound (US), particularly high-resolution gray-scale imaging combined with color Doppler (CD), has emerged as the primary imaging modality for evaluating acute scrotal conditions due to its accessibility, non-invasiveness, and high diagnostic accuracy.

Aims and objectives

This study aims to assess the diagnostic accuracy of US in differentiating the causes of acute scrotum, specifically focusing on testicular torsion, epididymo-orchitis, and varicocele. We also evaluate its role in reducing unnecessary surgical explorations and improving clinical decision-making in urgent settings.

Materials and methods

This retrospective observational study was conducted in a tertiary care center in Puducherry, India. A total of 250 male patients, ranging in age from 10 to 70 years, presented with acute scrotal pain over the 12-month study period. Inclusion criteria included all patients with sudden scrotal pain, regardless of clinical suspicion of underlying pathology. US scans were performed using a 7.5- to 12-MHz linear transducer. Gray-scale imaging was used to evaluate the testes, epididymis, spermatic cord, scrotal wall, and inguinal region, while CD was used to assess vascular flow. Statistical analysis was conducted using SPSS Version 18 (IBM Corp., Armonk, NY). Diagnostic accuracy was calculated based on sensitivity, specificity, positive predictive value, and negative predictive value.

Results

The most common cause of acute scrotum was inflammatory pathology, including epididymo-orchitis, accounting for 56.4% of cases (141 patients). Varicocele was the second most common cause, diagnosed in 11.6% of patients (29 cases), while hernias and epididymal cysts were found in 8% and 7.6% of cases, respectively. Testicular torsion was diagnosed in 2% of cases (five patients). US demonstrated high diagnostic accuracy for inflammatory pathologies, with sensitivity and specificity reaching 97% and 96%, respectively. The sensitivity for testicular torsion was 62%, reflecting the challenges in diagnosing partial or intermittent torsion, while specificity was high at 99%. US's performance in identifying varicocele was excellent, with a sensitivity of 93% and specificity of 98%.

Conclusion

US, especially when combined with CD, is an indispensable tool in the emergency evaluation of acute scrotum. It provides high diagnostic accuracy for inflammatory pathologies and varicocele while serving as an effective screening modality for testicular torsion. This study reinforces the role of US in reducing unnecessary surgical explorations, guiding clinical management, and improving patient outcomes in urgent clinical settings.

## Introduction

Acute scrotum represents a medical emergency, particularly in children and young adults, and is characterized by the sudden onset of scrotal pain, swelling, and occasionally redness. It is imperative to rapidly determine the underlying cause, as certain conditions such as testicular torsion necessitate urgent surgical intervention to preserve testicular viability. Other conditions, including epididymo-orchitis, are managed conservatively but still require a prompt and accurate diagnosis to mitigate long-term complications [1].

The differential diagnosis of acute scrotum encompasses a broad spectrum of conditions, ranging from benign self-limiting disorders to severe surgical emergencies. Common etiologies include inflammatory conditions such as epididymitis, epididymo-orchitis, testicular trauma, torsion of the testis or testicular appendage, and, in rare cases, testicular neoplasms. Among these, testicular torsion stands out as the most critical, with a narrow window of opportunity for surgical salvage [1,2]. The ability to distinguish between torsion and non-torsion causes of scrotal pain is crucial, as delayed intervention can result in irreversible testicular damage within 6-8 hours, significantly lowering the chances of preserving testicular function.

Clinical examination alone often fails to differentiate between the causes of acute scrotum, as the symptoms can overlap, and physical examination by visual inspection, palpation, and eliciting signs such as cremasteric reflux may be inconclusive due to the patient’s pain and discomfort [2,3]. Laboratory investigations such as urine dipstick, urine culture, and microscopy can only assist in clinical decision-making [4]. In this context, imaging plays an indispensable role, with ultrasound (US) emerging as the gold standard for evaluating acute scrotal conditions due to its rapid diagnostic capabilities. High-resolution gray-scale US, combined with color Doppler (CD), provides detailed anatomical and vascular information, facilitating rapid and accurate diagnosis [5].

US is non-invasive, widely available, cost-effective, and free of ionizing radiation, making it ideal for use in both emergency and outpatient settings. Gray-scale US allows for the visualization of the testicular parenchyma, epididymis, and spermatic cord, identifying structural abnormalities. CD US, on the other hand, evaluates testicular blood flow, which is particularly critical in diagnosing torsion and differentiating it from inflammatory conditions such as epididymo-orchitis, which typically show increased blood flow.

Advances in US technology, such as tissue harmonic imaging and the use of power Doppler, have further enhanced the sensitivity and specificity of US in diagnosing scrotal pathologies. The "whirlpool sign," observed on Doppler, is pathognomonic of spermatic cord torsion, while absent or decreased testicular blood flow is indicative of torsion requiring urgent surgical intervention.

Despite the clear benefits of US in diagnosing acute scrotum, the literature highlights varying rates of diagnostic accuracy across different settings and patient populations [5,6]. This variability underscores the need for context-specific studies to validate the effectiveness of US in different clinical environments.

The purpose of this study is to evaluate the role of high-resolution gray-scale US and CD in identifying and differentiating the various causes of acute scrotum in patients presenting to the emergency department of our tertiary care hospital. By establishing the diagnostic accuracy of US in this context, we aim to improve early detection of testicular torsion, optimize patient outcomes, and reduce unnecessary surgical interventions for non-torsion-related conditions.

Aims and objectives

The primary aim of this study is to evaluate the efficacy and diagnostic accuracy of high-resolution gray-scale US and CD in identifying and differentiating the underlying causes of acute scrotum. Specifically, the study aims to assess the role of US as a first-line imaging modality in distinguishing between surgical and non-surgical conditions such as testicular torsion, epididymo-orchitis, varicocele, hernias, and other less common etiologies.

The objective of this study is to assess the diagnostic accuracy of US in acute scrotum, determine the prevalence of various causes of acute scrotum, correlate US findings with clinical and surgical outcomes, identify patterns of age and etiology in acute scrotum, evaluate the role of US in avoiding unnecessary surgical exploration, and enhance clinical decision-making through early diagnosis.

## Materials and methods

Study design

This study is a retrospective observational study conducted at the Department of Radiodiagnosis, Mahatma Gandhi Medical College and Research Institute, Puducherry, India. The study aimed to evaluate the role of high-resolution US combined with CD in diagnosing the causes of acute scrotum in patients presenting to the emergency and outpatient departments. The study spanned a period of 12 months and included a sample size of 250 patients. IRB/IEC approval was waived off as the study was a retrospective observational analysis, using previously collected data without direct involvement or intervention with participants.

Study population

Inclusion Criteria

Male patients of all age groups presenting with acute scrotal pain, swelling, or tenderness, with or without additional systemic symptoms, were included.

Exclusion Criteria

Patients with a history of scrotal trauma or who presented with a known scrotal mass prior to the onset of pain were excluded. Patients with incomplete follow-up or those who did not consent to follow-up were also excluded from the final analysis.

Ultrasound machine

The study was conducted using a high-resolution US machine, specifically the GE Logiq P3 machine, equipped with linear 7.5- to 12-MHz transducers for optimal visualization of scrotal anatomy. Power Doppler and CD modes were used to assess vascularity and blood flow within the testes, epididymis, spermatic cord, and surrounding structures.

Imaging protocol

Patients were placed in a supine position with the scrotum supported by a towel or rolled-up cloth to ensure optimal exposure of the scrotal sac during imaging. In cases of suspected varicocele, the Valsalva maneuver was performed, and imaging was repeated in both supine and standing positions.

The scrotal contents were first evaluated using high-resolution gray-scale imaging in both longitudinal and transverse planes. This allowed for a detailed assessment of the testicular parenchyma, epididymis, and spermatic cord. Doppler imaging was employed to assess blood flow and vascularity, particularly to identify testicular torsion (reduced or absent blood flow) and inflammatory conditions (increased blood flow).

The testes were evaluated for size, echo-texture, and the presence of any lesions, masses, or cysts. Epididymis was assessed for enlargement, cyst formation, or other abnormalities. Spermatic cord was examined for signs of torsion (e.g., whirlpool sign), inflammation, or edema. Scrotal wall was checked for thickening or edema. The inguinal region was evaluated for the presence of hernia or other abnormalities. The kidney, ureter, and bladder (KUB) region was assessed to rule out referred pain from ureteral stones or other genitourinary pathologies causing secondary scrotal pain.

Data collection

Clinical data, including patient demographics, presenting symptoms, and clinical findings, were collected at the time of US examination. US findings were recorded for each patient, including details of gray-scale and Doppler findings. Follow-up data, including clinical outcomes and surgical findings (if any), were collected to correlate with the US diagnosis.

Statistical analysis

Data were analyzed using SPSS Version 18 for Windows (IBM Corp., Armonk, NY). Descriptive statistics, including means, frequencies, and percentages, were calculated for age distribution, presenting symptoms, and US findings. Sensitivity, specificity, positive predictive value, and negative predictive value were calculated to assess the accuracy of US in diagnosing the various causes of acute scrotum. Correlation between US findings and surgical or follow-up outcomes was analyzed to evaluate the diagnostic effectiveness of the imaging modality.

Sample size

A total of 250 patients were included in the final analysis, with an age range of 10 to 70 years. The demographic age distribution is summarized in Table 1.

**Table 1:** Age distribution of population presenting with scrotal symptoms.

Age (years)	Frequency	Percentage
10-20	22	8.8%
21-30	33	13.2%
31-40	41	16.4%
41-50	43	17.2%
51-60	57	22.8%
61-70	54	21.6%
Total	250	100%

## Results

The mean age was approximately 44.7 years. The most common age group affected by acute scrotum was 51 to 70 years, comprising 22.8% of the total cases.

Right-sided scrotal pain was the most common presentation, observed in 40% of patients (100 cases), as illustrated in Table 2. Bilateral involvement was seen in 26% of patients (65 cases), highlighting cases in which bilateral pathologies such as varicocele or systemic inflammatory responses were present.

**Table 2:** Side of involvement in scrotal pain

Side	Frequency	Percentage
Bilateral	65	26%
Left	85	34%
Right	100	40%
Total	250	100%

The most common causes of acute scrotum identified through US and CD are illustrated in Table 3. The study on the etiology of acute scrotum identified several key causes through US and CD evaluations. Epididymo-orchitis emerged as the predominant cause, with patients showing increased blood flow in the epididymis and testes, often accompanied by swelling and tenderness. Varicocele was the second most common cause, usually left-sided, where dilated veins in the scrotum showed retrograde blood flow, often becoming symptomatic after physical exertion. Inguinoscrotal hernias were another significant cause, with US detecting bowel or omental tissue in the scrotum, while CD ruled out strangulation. Epididymal cysts were the fourth most common finding, which were benign, fluid-filled lesions identified with no blood flow on Doppler. Other conditions included testicular torsion and testicular tumors. Some cases involved referred pain from a distal ureteral stone, with normal scrotal findings on US. In a few cases, no significant pathology was found, and the pain was managed conservatively.

**Table 3:** Etiological distribution of acute scrotum

Etiology	Frequency	Percentage
Distal ureteric calculus	6	2.4%
Epididymal cyst	19	7.6%
Hernia	20	8%
Inflammatory pathology	141	56.4%
Normal	24	9.6%
Testicular torsion	5	2%
Tumor	6	2.4%
Varicocele	29	11.6%
Total	250	100%

The correlation between age and specific etiologies showed that epididymo-orchitis was most prevalent in the age group of 51-60 years, with 38 out of 57 cases in this age group having this condition. Testicular torsion was primarily observed in younger patients, with cases distributed between 10 and 30 years. Varicocele was more common in middle-aged men, particularly between 31 and 50 years.

The diagnostic accuracy of US and CD in the evaluation of acute scrotum is illustrated in Table 4. This table reflects the performance of US in diagnosing various acute scrotal conditions across multiple studies, showing consistent results in sensitivity and specificity across the literature.

**Table 4:** Comparative table of sensitivity, specificity, PPV, and NPV for acute scrotal conditions The sensitivity and specificity of various acute scrotal pathologies are listed from Sharma et al. and Agarwal et al. [1,5]. Levenson et al. highlighted the use of US in detecting epididymo-orchitis with a sensitivity of nearly 100% and specificity of 95% [6]. Frohlich et al. reported 97% sensitivity and 100% specificity for diagnosing testicular torsion [7]. Demers and Pelsser’s study indicated that US's sensitivity and specificity for diagnosing varicocele was around 94% and 97% [8]. PPV, positive predictive value; NPV, negative predictive value; US, ultrasound

Condition	Current study				Sharma et al.		Agarwal et al.		Others	
	Sensitivity	Specificity	PPV	NPV	Sensitivity	Specificity	Sensitivity	Specificity	Sensitivity	Specificity
Calculus	75%	99%	75%	99%	78%	98%	77%	97%	90%	96%
Epididymal cyst	86%	98%	86%	98%	84%	96%	83%	95%	88%	95%
Hernia	91%	99%	91%	99%	89%	99%	88%	98%	92%	98%
Inflammatory pathology	97%	96%	97%	95%	95%	94%	96%	93%	100%	95%
Testicular torsion	62%	99%	83%	99%	65%	98%	64%	97%	97%	100%
Tumor	85%	99%	85%	99%	80%	98%	82%	98%	85%	99%
Varicocele	93%	98%	90%	99%	92%	97%	91%	96%	94%	97%

Clinical outcomes

Patients diagnosed with testicular torsion underwent immediate surgery, and the testis was successfully salvaged in the case of torsion. Cases of epididymo-orchitis were treated with antibiotics and anti-inflammatory medications, with resolution of symptoms in follow-up. Varicoceles and epididymal cysts were managed conservatively, with no need for surgical intervention unless symptoms worsened. Patients with hernia were referred to surgery, and the majority of cases were resolved without complications.

## Discussion

Acute scrotum is a broad term encompassing several urological emergencies that present with sudden onset of scrotal pain and swelling. Early and accurate diagnosis is critical in preventing irreversible damage, particularly in cases such as testicular torsion, where timely surgical intervention can preserve testicular function. US, specifically high-resolution gray-scale imaging combined with CD, is widely regarded as the gold standard for diagnosing acute scrotum, providing essential real-time information about testicular parenchyma and vascularity [9,10]. This study highlights the diagnostic accuracy of US and its clinical relevance, aligning with findings from a broad range of existing literature.

Testicular torsion is one of the most time-sensitive urological emergencies, and its diagnosis hinges largely on imaging modalities such as US. The ability of Doppler US to detect the absence of blood flow in torsion cases makes it an indispensable tool. In this study, US showed a sensitivity and specificity of 62% and 99%, respectively, for diagnosing testicular torsion, which is consistent with the findings of Pepe et al. and Blaivas et al., both of whom reported similar high diagnostic accuracy [11,12].

**Figure 1:**
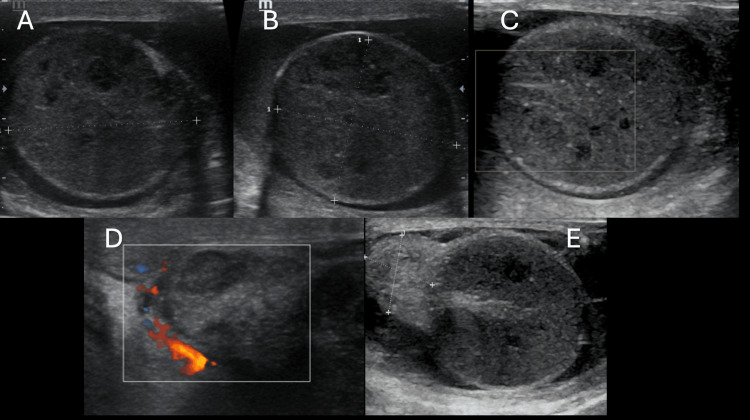
Testicular Torsion Figures 1A, 1B shows left side testicular enlargement, which appears edematous and heterogenous in architecture with multiple hypo-echoic spaces within. Figure 1C shows no evidence of vascularity on color Doppler. Figure 1D shows twisting of the spermatic cord noted above the level of testis. Figure 1E shows an enlarged and heterogenous epididymis.

Testicular torsion occurs when the spermatic cord twists, cutting off the blood supply to the testis. Studies have shown that the chance of saving the testis drops from nearly 100% within the first six hours to around 20% after 12 hours [13]. The ability of US to diagnose torsion promptly ensures that surgical detorsion can be performed in a timely manner, reducing the risk of testicular loss. The presence of the “whirlpool sign,” an indication of spermatic cord twisting, further supports a torsion diagnosis, as shown in the study by Vijayaraghavan [14]. The findings of Testicular torsion are illustrated in Figure 1.

The introduction of power Doppler and spectral Doppler techniques has further enhanced the diagnostic capabilities of US. These modalities allow for a more detailed assessment of blood flow, particularly in cases of partial or intermittent torsion, which can present a diagnostic challenge [10,15].

Dogra et al. emphasized the importance of spectral Doppler in identifying diminished diastolic flow, which can differentiate partial torsion from epididymo-orchitis [16].

Epididymo-orchitis, an inflammatory condition of the epididymis and testis, was the most common cause of acute scrotum in this study, accounting for 56% of cases. This aligns with findings from Agrawal et al. and Demers and Pelsser, who also reported a high prevalence of inflammatory pathologies in their respective studies [5,8]. In our study, US demonstrated a sensitivity of 97% and specificity of 96% for epididymo-orchitis, which is consistent with previously published data.

**Figure 2:**
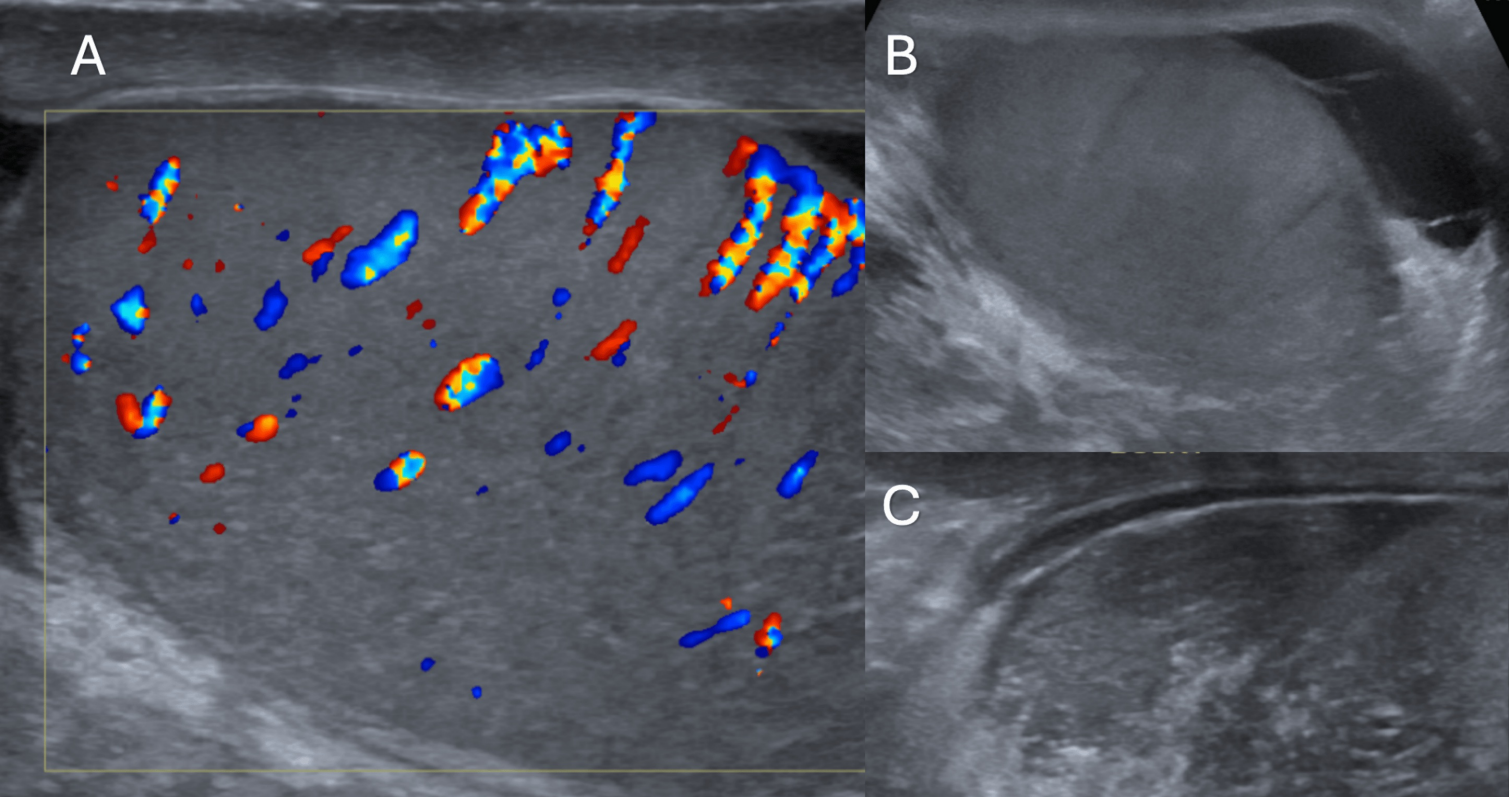
Epididymo-orchitis Figure 2A shows a bulky right testis, with a heterogenous echo pattern with increased vascularity on color Doppler. Figure 2B shows mild hydrocele with few thin internal septations. Figure 2C shows a bulky and heterogenous epididymis.

CD plays a critical role in differentiating epididymo-orchitis from testicular torsion. In epididymo-orchitis, increased vascularity due to inflammation, as illustrated in Figure 2, is seen on Doppler as hyperemia in the epididymis and testis [6]. In contrast, torsion typically shows a lack of blood flow, which allows clinicians to make a rapid and accurate diagnosis. Singh et al. emphasized the utility of CD in distinguishing between these two conditions, as both can present similarly with scrotal pain and swelling [17].

The pathophysiology of epididymo-orchitis is typically related to ascending urinary tract infections, particularly in older patients. Our study showed a higher prevalence of this condition in men aged 40-50 years, which is consistent with other studies. Early diagnosis using US helps guide appropriate management, typically involving antibiotics, and avoids unnecessary surgical exploration [8].

**Figure 3:**
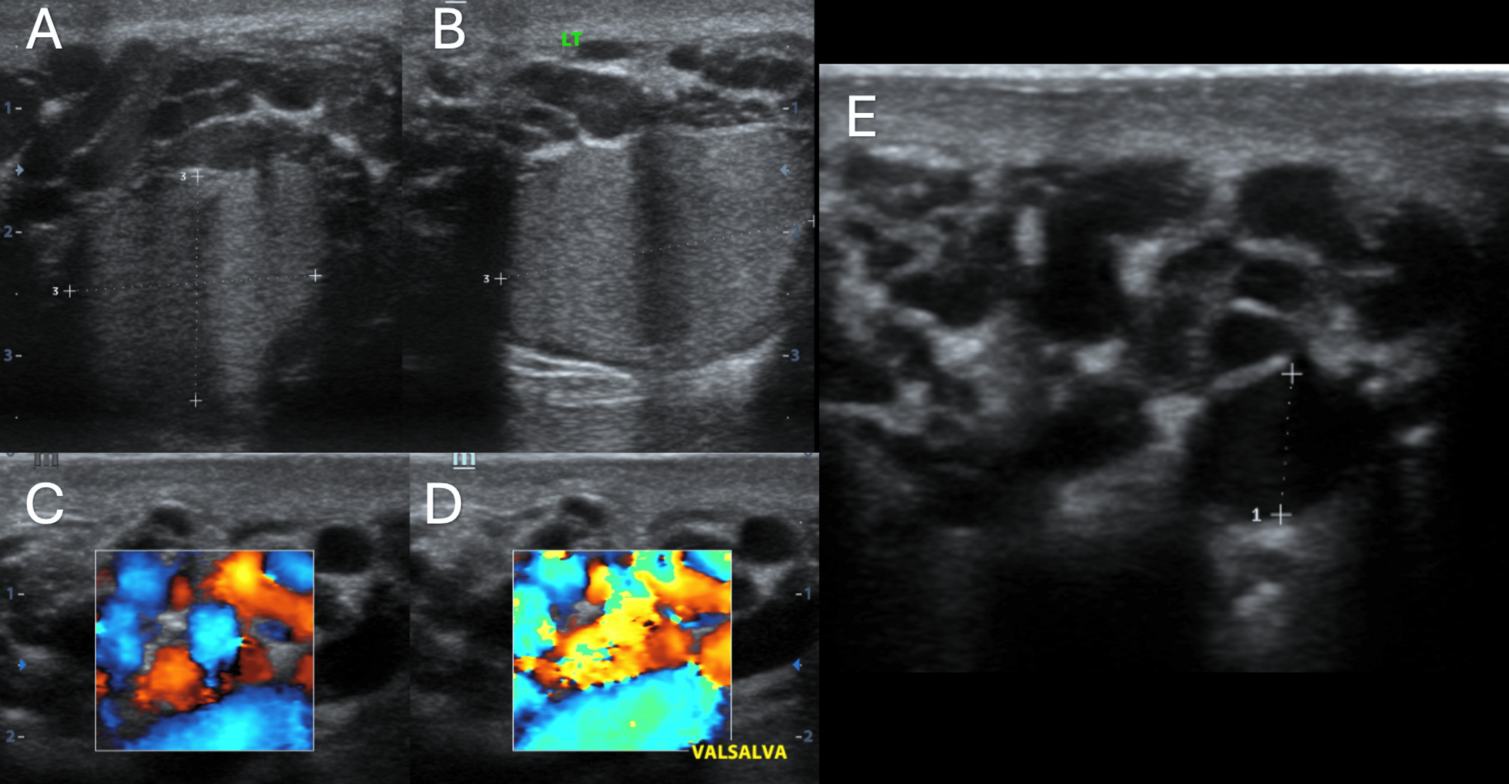
Grade IV varicocele Figures 3A, 3B (transverse and longitudinal scan of left scrotum) show multiple dilated and tortuous pampiniform plexus. Figures 3C, 3D (CD images at rest and on Valsalva) show spontaneous reflux on the Valsalva maneuver. Figure 3E shows the dilated vein with a maximum caliber of 8 mm.

Varicocele was the second most common cause of acute scrotum in this study, accounting for 12% of cases. Even though varicocele presents more frequently with a dull aching or throbbing pain, sometimes it can cause a sharp, acute, or stabbing pain [18,19]. Varicoceles are characterized by dilated veins in the pampiniform plexus, and they can cause discomfort, particularly after prolonged standing or physical exertion. US, particularly when combined with the Valsalva maneuver, enables accurate detection of varicocele by demonstrating venous reflux (Figure 3). Several studies, including those by Valentino et al. and Lee et al., have demonstrated the high sensitivity and specificity of Doppler US in detecting varicocele [20,21].

Our study showed similar findings, with a sensitivity of 93% and specificity of 98% for diagnosing varicocele. This highlights the utility of US not only in diagnosing varicocele but also in distinguishing it from other causes of scrotal swelling, such as hydrocele or hernia.

**Figure 4:**
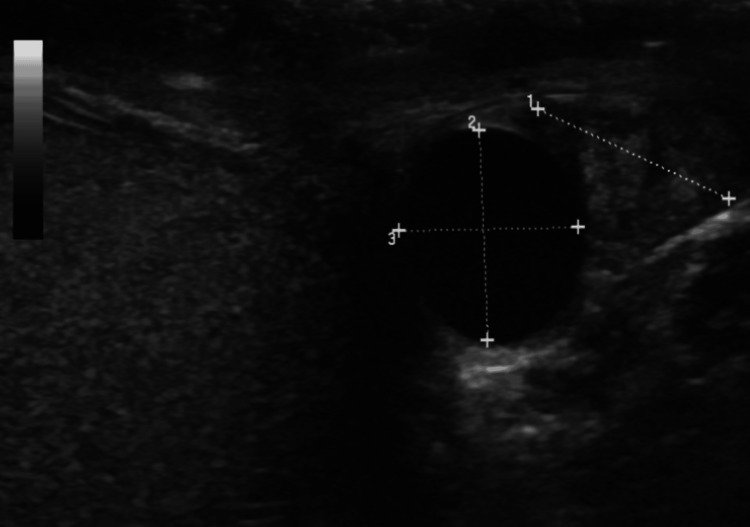
Epididymal cyst. An anechoic cystic lesion is noted in the head of epididymis.

Inguinoscrotal hernias and epididymal cysts accounted for 8% and 7.6% of cases, respectively, in this study. Inguinal hernias, particularly those that extend into the scrotum, can be visualized on US as bowel loops or omentum within the scrotal sac. The study by Prajapati et al. has emphasized the role of US in diagnosing these hernias, especially in ruling out complications such as strangulation [22]. Epididymal cysts, on the other hand, present as well-circumscribed, anechoic lesions within the epididymis on gray-scale US, with no associated blood flow on Doppler (Figure 4). While these cysts are typically benign and asymptomatic, accurate diagnosis is important to differentiate them from more concerning pathologies such as tumors.

**Figure 5:**
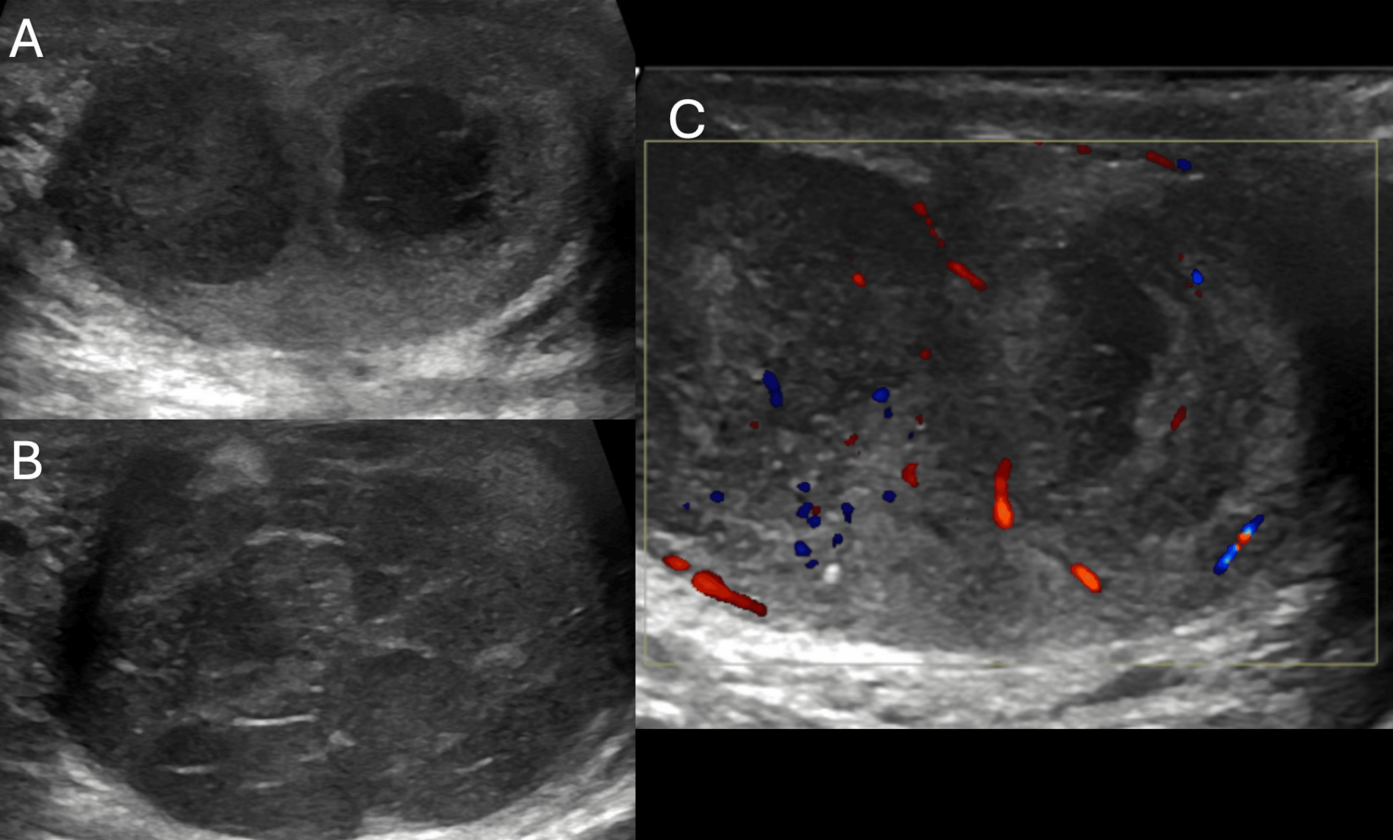
Testicular tumor Figures 5A, 5B show that the testis is replaced with diffuse heterogenous lesion, resulting in distortion of testicular architecture and enlarged appearance. Figure 5C shows areas of internal vascularity on color Doppler.

The diagnosis of a testicular tumor highlights the importance of US in detecting rare causes of acute scrotum. Testicular tumors typically present as solid, hypoechoic masses on gray-scale US, with no associated blood flow on Doppler. The findings of testicular tumor are illustrated in Figure 5. The role of US in the early detection of testicular tumors cannot be understated, as early diagnosis significantly improves prognosis. Dogra et al. and Altinkilic et al. have both reported the efficacy of US in identifying testicular tumors, particularly when combined with Doppler [16,23].

Clinical relevance and impact

The findings of this study emphasized the clinical utility of US in the diagnosis of acute scrotum. By providing accurate, real-time imaging, US allows for early differentiation between surgical emergencies, such as testicular torsion, and conditions that can be managed conservatively, such as epididymo-orchitis or varicocele. This reduces the need for unnecessary surgeries and ensures that patients receive appropriate treatment promptly.

In clinical practice, US should be the first-line imaging modality for all patients presenting with acute scrotum, as it is non-invasive, is widely available, and provides essential diagnostic information. Studies by Frohlich et al. and Baker et al. have shown that the use of US in emergency settings significantly reduces the need for exploratory surgery by accurately ruling out torsion [7,24].

Limitations of the study

While this study provides valuable insights, it is limited by its relatively small sample size of 250 patients. Larger studies, ideally conducted across multiple centers, would provide more robust data and allow for better generalization of the findings. Additionally, although US is highly effective, as highlighted by Cassar et al., there remains a risk of misdiagnosis in cases of intermittent or partial torsion [25].

Future directions

Future research should explore the role of advanced US techniques, such as contrast-enhanced US, which has shown promise in improving diagnostic accuracy for acute scrotal conditions. Additionally, elastography, which assesses tissue stiffness, may offer further diagnostic advantages, particularly in differentiating benign from malignant lesions.

## Conclusions

US, particularly high-resolution gray-scale imaging combined with CD, has proven to be an indispensable tool in the evaluation of acute scrotum, enabling rapid and accurate differentiation between surgical and non-surgical causes of scrotal pain. In this study, US demonstrated exceptional sensitivity and specificity in diagnosing various conditions and underscores its role as the first-line imaging modality for scrotal emergencies, where early intervention can prevent testicular loss. The ability of US to reduce unnecessary surgical explorations while ensuring timely treatment for emergencies such as testicular torsion highlights its clinical significance. By providing real-time, non-invasive assessment, US improves patient outcomes and minimizes the risk of complications associated with delayed or incorrect diagnoses.

In conclusion, US remains the gold standard for evaluating acute scrotum, playing a pivotal role in both emergency and outpatient settings by facilitating rapid diagnosis, guiding appropriate management, and improving overall patient care.
